# (*E*)-3-Dimethyl­amino-1-(2-thien­yl)prop-2-en-1-one

**DOI:** 10.1107/S1600536809025331

**Published:** 2009-07-11

**Authors:** Jian-Hong Bi

**Affiliations:** a Deparment of Chemistry and Chemical Engineering, Hefei Teachers College, Hefei 230061, People’s Republic of China

## Abstract

The mol­ecular skeleton of the title mol­ecule, C_9_H_11_NOS, is essentially planar: the thio­phene ring is inclined to the mean plane of the rest non-H atoms by 2.92 (3)°. The crystal packing exhibits no significantly short inter­molecular contacts.

## Related literature

For general backgroud, see Amari *et al.* (1993 ▶). For the crystal structures of related compounds, see: Li *et al.* (2005 ▶); Hu *et al.* (2007 ▶); Bi (2009 ▶).
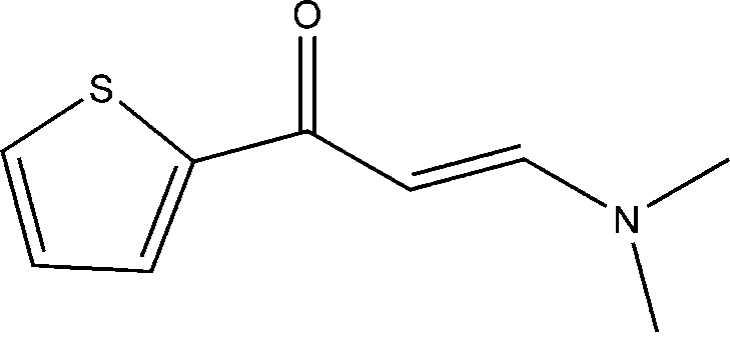


## Experimental

### 

#### Crystal data

C_9_H_11_NOS
*M* *_r_* = 181.26
Monoclinic, 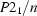

*a* = 5.9618 (12) Å
*b* = 8.1241 (16) Å
*c* = 19.449 (4) Å
β = 92.910 (3)°
*V* = 940.8 (3) Å^3^
*Z* = 4
Mo *K*α radiation
μ = 0.30 mm^−1^
*T* = 291 K
0.45 × 0.30 × 0.15 mm

#### Data collection

Bruker SMART CCD area-detector diffractometer
Absorption correction: multi-scan (*SADABS*; Bruker, 2000 ▶) *T*_min_ = 0.867, *T*_max_ = 0.964
4740 measured reflections
1636 independent reflections
1137 reflections with *I* > 2σ(*I*)
*R*_int_ = 0.034

#### Refinement

*R*[*F* ^2^ > 2σ(*F* ^2^)] = 0.047
*wR*(*F* ^2^) = 0.124
*S* = 1.05
1636 reflections
111 parameters
H-atom parameters constrained
Δρ_max_ = 0.20 e Å^−3^
Δρ_min_ = −0.29 e Å^−3^

### 

Data collection: *SMART* (Bruker, 2000 ▶); cell refinement: *SAINT* (Bruker, 2000 ▶); data reduction: *SAINT*; program(s) used to solve structure: *SHELXTL* (Sheldrick, 2008 ▶); program(s) used to refine structure: *SHELXTL*; molecular graphics: *SHELXTL*; software used to prepare material for publication: *SHELXTL*.

## Supplementary Material

Crystal structure: contains datablocks I, global. DOI: 10.1107/S1600536809025331/cv2582sup1.cif

Structure factors: contains datablocks I. DOI: 10.1107/S1600536809025331/cv2582Isup2.hkl

Additional supplementary materials:  crystallographic information; 3D view; checkCIF report

## Figures and Tables

**Fig. 1.**
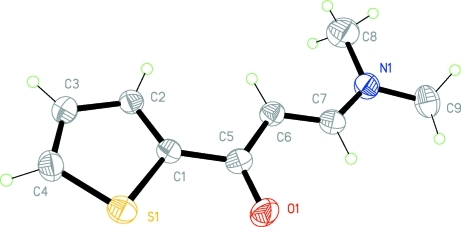
Molecular structure of (I) showing the atomic numbering and 30% probabilty displacement ellipsoids.

## supplementary crystallographic information

### Comment

Many coordinated complexes derived from 2-[3-(dimethylamino)prop-2-enoyl] pyridine or thiophene have been reported recently in chemical research (Amari *et al.*, 1993; Bi, 2009; Hu & Tian, 2007; Li *et al.*, 2005). In continuation of our ongoing program directed to the development of similar compounds (Bi, 2009), herein we report the molecular structure of the title compound (I) - the newly synthesized ligand derived from 2-acetylthiophene.

In (I) (Fig. 1), the dihedral angle between the thiophene ring and the mean plane of the restnon-hydrogen atoms is  2.92 (3)°. The crystal packing exhibits no significantly short intermolecular contacts.

### Experimental

All solvents and chemicals were of analytical grade and were used without further purification. A solution of 2-acetylthiophene (0.2 mmol) and dimethoxy-*N*,*N*-dimethylmethanamine(0.2 mmol) in 150 ml DMF was refluxed for 8 h, and then concentrated to give the title compound. Single crystals suitable for X-ray analysis were grown from the methanol solution by slow evaporation at room temperature in air. Anal. Calcd.for C_9_H_11_NOS: C, 59.64; H, 6.12; N, 7.73. Found: C, 39.65; H,6.16; N, 7.71.

### Refinement

All hydrogen atoms were geometrically positioned (C—H 0.93–0.97 Å) and refined as riding, with *U*_iso_(H)=1.2–1.5 *U*_eq_ of the parent atom.

### Crystal data

**Table:**

C_9_H_11_NOS	*F*(000) = 384
*M**_r_* = 181.26	*D*_x_ = 1.280 Mg m^−^^3^
Monoclinic, *P*2_1_/*n*	Mo *K*α radiation, λ = 0.71073 Å
Hall symbol: -P 2yn	Cell parameters from 955 reflections
*a* = 5.9618 (12) Å	θ = 2.7–20.2°
*b* = 8.1241 (16) Å	µ = 0.30 mm^−^^1^
*c* = 19.449 (4) Å	*T* = 291 K
β = 92.910 (3)°	Block, yellow
*V* = 940.8 (3) Å^3^	0.45 × 0.30 × 0.15 mm
*Z* = 4	

### Data collection

**Table:**

Bruker SMART CCD area-detector diffractometer	1636 independent reflections
Radiation source: fine-focus sealed tube	1137 reflections with *I* > 2σ(*I*)
graphite	*R*_int_ = 0.034
φ and ω scans	θ_max_ = 25.0°, θ_min_ = 2.1°
Absorption correction: multi-scan (*SADABS*; Bruker, 2000)	*h* = −7→6
*T*_min_ = 0.867, *T*_max_ = 0.964	*k* = −9→8
4740 measured reflections	*l* = −23→19

### Refinement

**Table:**

Refinement on *F*^2^	Primary atom site location: structure-invariant direct methods
Least-squares matrix: full	Secondary atom site location: difference Fourier map
*R*[*F*^2^ > 2σ(*F*^2^)] = 0.047	Hydrogen site location: inferred from neighbouring sites
*wR*(*F*^2^) = 0.124	H-atom parameters constrained
*S* = 1.05	*w* = 1/[σ^2^(*F*_o_^2^) + (0.0547*P*)^2^ + 0.2232*P*] where *P* = (*F*_o_^2^ + 2*F*_c_^2^)/3
1636 reflections	(Δ/σ)_max_ < 0.001
111 parameters	Δρ_max_ = 0.20 e Å^−^^3^
0 restraints	Δρ_min_ = −0.29 e Å^−^^3^

### Special details

**Table:**

Experimental. The structure was solved by direct methods (Bruker, 2000) and successive difference Fourier syntheses.
Geometry. All e.s.d.'s (except the e.s.d. in the dihedral angle between two l.s. planes) are estimated using the full covariance matrix. The cell e.s.d.'s are taken into account individually in the estimation of e.s.d.'s in distances, angles and torsion angles; correlations between e.s.d.'s in cell parameters are only used when they are defined by crystal symmetry. An approximate (isotropic) treatment of cell e.s.d.'s is used for estimating e.s.d.'s involving l.s. planes.
Refinement. Refinement of *F*^2^ against ALL reflections. The weighted *R*-factor *wR* and goodness of fit *S* are based on *F*^2^, conventional *R*-factors *R* are based on *F*, with *F* set to zero for negative *F*^2^. The threshold expression of *F*^2^ > σ(*F*^2^) is used only for calculating *R*-factors(gt) *etc*. and is not relevant to the choice of reflections for refinement. *R*-factors based on *F*^2^ are statistically about twice as large as those based on *F*, and *R*- factors based on ALL data will be even larger.

### Fractional atomic coordinates and isotropic or equivalent isotropic displacement parameters (Å^2^)

**Table:**

	*x*	*y*	*z*	*U*_iso_*/*U*_eq_	
C1	0.0234 (4)	0.4667 (3)	0.16370 (13)	0.0425 (6)	
C2	0.2349 (4)	0.5260 (3)	0.18109 (14)	0.0462 (7)	
H2	0.3586	0.5108	0.1545	0.055*	
C3	0.2416 (5)	0.6127 (3)	0.24415 (16)	0.0581 (8)	
H3	0.3707	0.6620	0.2635	0.070*	
C4	0.0404 (5)	0.6165 (4)	0.27337 (15)	0.0594 (8)	
H4	0.0155	0.6685	0.3149	0.071*	
C5	−0.0592 (4)	0.3689 (3)	0.10366 (14)	0.0479 (7)	
C6	0.0996 (4)	0.3228 (3)	0.05454 (14)	0.0481 (7)	
H6	0.2501	0.3510	0.0618	0.058*	
C7	0.0307 (4)	0.2381 (3)	−0.00247 (14)	0.0499 (7)	
H7	−0.1220	0.2142	−0.0066	0.060*	
C8	0.3912 (5)	0.2183 (5)	−0.05347 (19)	0.0906 (12)	
H8A	0.4146	0.3350	−0.0566	0.136*	
H8B	0.4532	0.1650	−0.0922	0.136*	
H8C	0.4638	0.1775	−0.0117	0.136*	
C9	0.0524 (6)	0.0980 (4)	−0.11220 (15)	0.0691 (9)	
H9A	−0.1052	0.0830	−0.1064	0.104*	
H9B	0.1233	−0.0074	−0.1162	0.104*	
H9C	0.0732	0.1611	−0.1531	0.104*	
N1	0.1517 (4)	0.1844 (3)	−0.05331 (12)	0.0566 (6)	
O1	−0.2623 (3)	0.3317 (3)	0.09894 (10)	0.0688 (6)	
S1	−0.15994 (12)	0.51590 (10)	0.22552 (4)	0.0598 (3)	

### Atomic displacement parameters (Å^2^)

**Table:**

	*U*^11^	*U*^22^	*U*^33^	*U*^12^	*U*^13^	*U*^23^
C1	0.0437 (14)	0.0414 (15)	0.0427 (15)	0.0011 (11)	0.0066 (12)	0.0028 (12)
C2	0.0414 (14)	0.0467 (16)	0.0509 (17)	−0.0023 (11)	0.0057 (12)	−0.0032 (13)
C3	0.0515 (16)	0.0588 (19)	0.0636 (19)	−0.0033 (14)	−0.0019 (14)	−0.0104 (16)
C4	0.0681 (19)	0.0601 (19)	0.0499 (18)	0.0025 (15)	0.0019 (15)	−0.0097 (15)
C5	0.0451 (15)	0.0498 (17)	0.0489 (17)	−0.0032 (12)	0.0040 (13)	0.0067 (14)
C6	0.0435 (14)	0.0520 (17)	0.0492 (17)	−0.0036 (12)	0.0065 (13)	0.0014 (14)
C7	0.0480 (16)	0.0532 (17)	0.0492 (17)	0.0003 (12)	0.0093 (14)	0.0046 (14)
C8	0.062 (2)	0.128 (3)	0.084 (3)	−0.002 (2)	0.0275 (19)	−0.016 (2)
C9	0.091 (2)	0.064 (2)	0.0531 (19)	−0.0050 (18)	0.0129 (17)	−0.0053 (17)
N1	0.0547 (14)	0.0666 (16)	0.0494 (14)	−0.0018 (12)	0.0109 (11)	−0.0068 (13)
O1	0.0444 (11)	0.0969 (16)	0.0658 (14)	−0.0154 (10)	0.0100 (10)	−0.0200 (12)
S1	0.0495 (5)	0.0741 (6)	0.0571 (5)	−0.0014 (4)	0.0145 (4)	−0.0052 (4)

### Geometric parameters (Å, °)

**Table:**

C1—C2	1.376 (3)	C6—H6	0.9300
C1—C5	1.476 (4)	C7—N1	1.327 (3)
C1—S1	1.713 (2)	C7—H7	0.9300
C2—C3	1.413 (4)	C8—N1	1.455 (4)
C2—H2	0.9300	C8—H8A	0.9600
C3—C4	1.353 (4)	C8—H8B	0.9600
C3—H3	0.9300	C8—H8C	0.9600
C4—S1	1.688 (3)	C9—N1	1.444 (4)
C4—H4	0.9300	C9—H9A	0.9600
C5—O1	1.247 (3)	C9—H9B	0.9600
C5—C6	1.429 (3)	C9—H9C	0.9600
C6—C7	1.351 (4)		
			
C2—C1—C5	130.4 (2)	N1—C7—H7	115.7
C2—C1—S1	110.8 (2)	C6—C7—H7	115.7
C5—C1—S1	118.81 (18)	N1—C8—H8A	109.5
C1—C2—C3	111.9 (2)	N1—C8—H8B	109.5
C1—C2—H2	124.0	H8A—C8—H8B	109.5
C3—C2—H2	124.0	N1—C8—H8C	109.5
C4—C3—C2	112.9 (3)	H8A—C8—H8C	109.5
C4—C3—H3	123.5	H8B—C8—H8C	109.5
C2—C3—H3	123.5	N1—C9—H9A	109.5
C3—C4—S1	112.0 (2)	N1—C9—H9B	109.5
C3—C4—H4	124.0	H9A—C9—H9B	109.5
S1—C4—H4	124.0	N1—C9—H9C	109.5
O1—C5—C6	124.1 (3)	H9A—C9—H9C	109.5
O1—C5—C1	118.2 (2)	H9B—C9—H9C	109.5
C6—C5—C1	117.7 (2)	C7—N1—C9	122.3 (2)
C7—C6—C5	119.9 (2)	C7—N1—C8	120.7 (3)
C7—C6—H6	120.1	C9—N1—C8	116.9 (2)
C5—C6—H6	120.1	C4—S1—C1	92.36 (13)
N1—C7—C6	128.7 (3)		

## References

[bb1] Amari, C., Ianelli, S., Pelizzi, C., Pelizzi, G. & Predieri, G. (1993). *Inorg. Chim. Acta*, **211**, 89–94.

[bb2] Bi, J.-H. (2009). *Acta Cryst.* E**65**, m633. 10.1107/S1600536809016845 PMC2969776 21583001

[bb3] Bruker (2000). *SADABS*, *SMART* and *SAINT* Bruker AXS Inc., Madison, Wisconsin, USA.

[bb4] Hu, T.-L. & Tian, J.-L. (2007). *Acta Cryst.* E**63**, m1092–m1093.

[bb5] Li, G.-X., Li, J.-Q. & Kang, X.-Z. (2005). *Acta Cryst.* E**61**, m410–m411.

[bb6] Sheldrick, G. M. (2008). *Acta Cryst.* A**64**, 112–122. 10.1107/S0108767307043930 18156677

